# A Neural Field Model of the Somatosensory Cortex: Formation, Maintenance and Reorganization of Ordered Topographic Maps

**DOI:** 10.1371/journal.pone.0040257

**Published:** 2012-07-12

**Authors:** Georgios Is. Detorakis, Nicolas P. Rougier

**Affiliations:** INRIA CNRS: UMR 7503 Université Henri Poincaré - Nancy I Université Nancy II Institut National Polytechnique de Lorraine, Nancy, France; University of Bologna, Italy

## Abstract

We investigate the formation and maintenance of ordered topographic maps in the primary somatosensory cortex as well as the reorganization of representations after sensory deprivation or cortical lesion. We consider both the critical period (postnatal) where representations are shaped and the post-critical period where representations are maintained and possibly reorganized. We hypothesize that feed-forward thalamocortical connections are an adequate site of plasticity while cortico-cortical connections are believed to drive a competitive mechanism that is critical for learning. We model a small skin patch located on the distal phalangeal surface of a digit as a set of 256 Merkel ending complexes (MEC) that feed a computational model of the primary somatosensory cortex (area 3b). This model is a two-dimensional neural field where spatially localized solutions (a.k.a. bumps) drive cortical plasticity through a Hebbian-like learning rule. Simulations explain the initial formation of ordered representations following repetitive and random stimulations of the skin patch. Skin lesions as well as cortical lesions are also studied and results confirm the possibility to reorganize representations using the same learning rule and depending on the type of the lesion. For severe lesions, the model suggests that cortico-cortical connections may play an important role in complete recovery.

## Introduction

Early observations of Leyton and Sherrington [1] (as reported by Lemon in [2]) on the adult anthropoid apes demonstrated the ability of the motor cortex to recover from extensive cortical lesions. The authors hypothesized consequently the existence of a neural substrate and/or a mechanism for such extensive recovery. However, about forty years later, Hubel and Wiesel published a very influential paper [3] that promoted the idea of fixed cortical representations following the post-natal developmental period. This hypothesis has prevailed for a long time until the studies of Merzenich, Kaas et al. [4]–[6] provided experimental evidence for somatosensory cortex reorganization following a peripheral nerve injury or amputation in the adult monkey. Several neurophysiological studies [7]–[9] have since confirmed this latter hypothesis and the cortex is now considered as a dynamic structure that is able to reorganize its representations during the whole life-time and not only during the *critical period*. It has been confirmed for the case of lesion (e.g. strokes) [9], [10], ablation [11], [12] (e.g. tumors surgery), body injury (e.g. accident) or severe degeneracy’s of thalamocortical and cortico-spinal projections [13], [14]. Even environmental factors may deeply impact cortical representations as it has been demonstrated by Daniel et al. in [15]. However, the nature of the underlying mechanisms supporting such cortical plasticity is still largely unknown even if some hypotheses have emerged. In this regard, Feldman and Brecht [16] published an extensive review of synaptic mechanisms that could be responsible for plasticity in the neocortex at both the *synaptic physiological* level (long-term potentiation (LTP), long-term depression (LTD), spike timing dependent plasticity (STDP), homeostasis, meta-plasticity, GABAergic cells and circuits) and the *structural* level (thalamocortical and horizontal cross-columnar axons). Hickmott and Merzenich [17] proposed a similar study about the properties of local circuit underlying cortical reorganization and identified two general classes of mechanisms, one involves *a rapid change in the efficiency of existing synapses* while the other entails *a delayed phase promoting the sprouting of new connections*. This latter study is in fact quite consistent with the former two-levels analysis. It is to be noted that some of these mechanisms were already hypothesized to be involved in cortical plasticity. For example, neuronal axon sprouting was also reported by Florence et al. [18] as a potential candidate, LTP and LTD in [19]–[22], formation of new synapses [4], [23] and inhibitory and excitatory mechanisms in [24], [25]. Some other candidates have been identified as well as the role of the inter hemispheric modulation of somatosensory receptive fields [26]. Today, no definitive hypothesis has emerged and most probably the answer is a combination of different mechanisms at different time scale proportionally to the considered period of development (prenatal/postnatal critical period/adult period).

One difficulty in identifying such a mechanism is that one must give account on both the initial formation of ordered topographic maps (as it has been observed in primary sensory areas V1, A1 and S1 for example), the maintenance of such maps during the whole lifetime, the reorganization following a trauma or an injury and the possible refinement according to experience (e.g. expanding representations in order to increase accuracy). Since the anatomical organization of the cortex follows a regular and hierarchical structure [27], [28] whose elementary circuit is the minicolumn (even if they may display considerable differences [29]), this latter structure or the macrocolumn (which gathers from 60 to 80 minicolumns [30]) may both represent natural candidates to be investigated further. More precisely, we know that each cortical layer receives input from four distinct sources, one from extra-cortical areas and three from intra-cortical areas. First, excitatory neurons of a single layer receive input from other neurons of the same layer. Second, excitatory neurons of the input layer, L4, receive recurrent feedback input from L2/3. Thus a positive recurrent loop emerges which seems to account for gain modulation for active selection and re-combination of the relatively small afferent signals [31]. Third is the background noise of the cortical circuit that has been proposed to contributes to the modulation of the gain of the circuit by enhancing the responsiveness of cortical pyramidal neurons, [32], [33]. Last, but not least, excitatory neurons of L4 receive direct input from the thalamus and if they account approximately for only 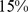 of the synapses, Bruno and Sakmann [34] demonstrated *in vivo* they may nonetheless drive the cortex without the need for intra-cortical amplification and revealed the thalamocortical pathway as a highly efficient one. In addition, thalamic neurons develop direct mono-synaptic connections onto L4 cortical excitatory neurons independently of the morphological characteristics of these neurons. Furthermore, Khazipov et al. [35] described the ontological development of the cortex and the respective contribution of different mechanisms. The maturation of the brain is divided in two major periods, pre- and postnatal. At the beginning of the prenatal period, genetic information leads to the early formation of the neural networks followed by a spontaneous electrical activity period that leads to the formation of the columnar organization [36], [37]. This process continues after birth and stops when the *critical* period starts. Then, cortical circuits are driven mainly by experience and synaptic plasticity (e.g. Hebbian learning) takes place. After the critical period comes to an end the adult brain can still learn and cortical circuits are able to reorganize themselves and refine their receptive fields.

At this point, we think that computational neuroscience may play a key role by providing computational models that can be used to test this or that functional hypothesis. It has been already the case with the self-organizing maps as proposed by T. Kohonen [38], [39] in the late eighties that helped to promote the idea of a competition among units leading to the formation of ordered representations (although without the possibility of re-organizing them). At the same time, G. Edelman was proposing to the community his theory of neural group selection [40] and more specifically, he was proposing a computational model of plasticity in the organization of the cortical maps [41] where neuronal *groups serve as the basic unit for map organization*. However, this model did not emphasize the importance of the thalamocortical pathway as we explained earlier and we think we might need to reconsider its role in the formation of and maintenance of the sensory representations.

We propose in this article to investigate (computationally) the formation of topographic maps in the somatosensory cortex as well as the reorganization of representations after sensory deprivation or cortical lesion. We consider both the critical period (postnatal) where representations are shaped and the post-critical period where representations are maintained and possibly reorganized. We hypothesize that feed-forward thalamocortical connections are an adequate site of plasticity to give account for both the formation and the maintenance of topographic representations and partly for the reorganization of representations following a sensory or cortical lesion. We therefore focus on the organization and reorganization of the somatosensory cortex (area 3b) innervated by the mechanoreceptors of the hand. A model of skin and its associated mechanoreceptors (Merkel ending complexes) is first introduced and the cortical model, based on the dynamic neural field theory, is presented together with its dynamics that allow to drive learning through a Hebbian-like learning rule. Results concerning the initial formation and maintenance of ordered representation are analyzed as well as results concerning the reorganization of representations following a cortical or skin lesion. In light of these experiments, we discuss the critical role of feed-forward thalamocortical connections in the reorganization process as well as the potential role of lateral connections.

## Methods

### Skin Model

**Figure 1:**
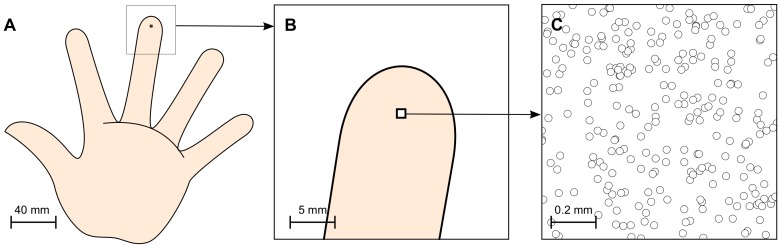
Skin modeling. **A** A palmar schematic of the hand ((https://commons.wikimedia.org/wiki/File:Hand_left.svg) Hand drawing by Cy21 available on commons.wikimedia.org under a Creative Commons Attribution-Share Alike 3.0 Unported https://creativecommons.org/licenses/by-sa/3.0/deed.enlicense.) **B** Location and relative size of the modeled skin patch. **C** Magnification of skin patch indicating the topology of receptors.



**Figure 2:**
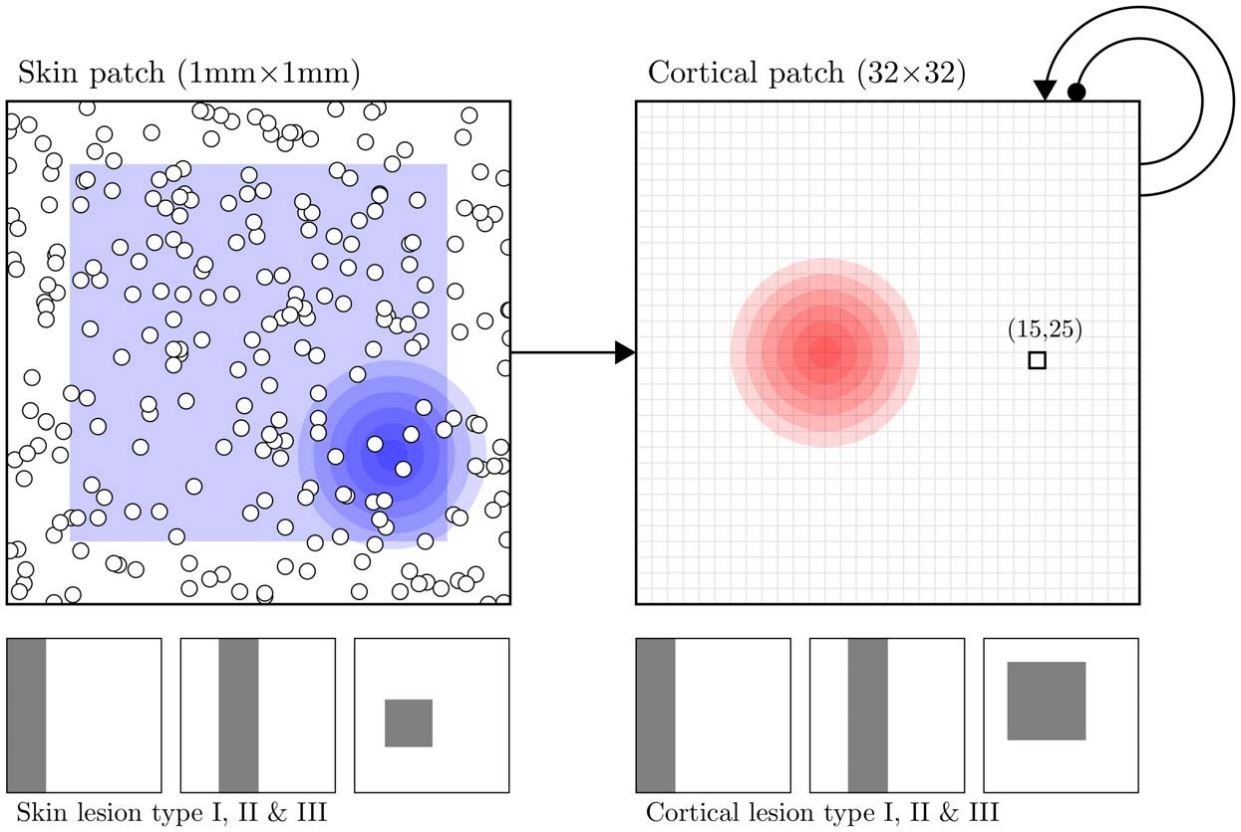
A model of the somatosensory cortex. The skin patch is modeled as a set of 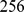 mechanic receptors (white discs in the figure) with a quasi-uniform distribution that feed the cortical patch. Blue circles represent an example of a stimulus applied on the skin patch and the blue square represents the stimulation area. The cortical patch is modeled using a neural field with a spatial discretization of size 32×32 elements using global lateral excitation and inhibition. Red circles represent a (schematic) typical cortical response after learning. The three squares under each patch represent the different cases of lesion that have been studied where the gray part represents the lesioned area.

We modeled the Merkel ending complexes (MEC) that are dedicated to sustained touch sensation and pressure, neglecting other modalities (e.g. temperature, pain). Following data provided by Pare et al. in [42], we considered a small skin patch located on the distal phalangeal surface of a digit (see figure 1) that accounts roughly for half of the digit surface in area 3b, rest of the surface being shared among proximal and middle surface of the same digit [43]. The skin patch is approximately of size 1 mm^2^, using a receptor density of 250/mm^2^ [44]. It has been modeled as a planar surface 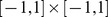 (arbitrary units) and we considered 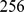 MEC’s that are arranged in a regular grid over the whole surface with a location jitter of 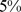. This results in a quasi-uniform distribution consistent with actual distribution of MEC as reported in [42] and illustrated in figure 1C. Each receptor is fully described by its Cartesian coordinates, namely 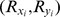, where 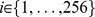. We assume that when a stimulus is applied at a given location 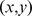 of the skin patch, the mechanic property of the skin extends the pressure level to nearby locations [45] such that the response 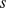 of any receptor (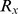, 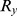) is given by:

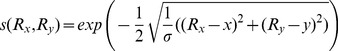
(1)In primates, this somatosensory information flows through several relays, which lie in the spinal cord and the thalamus, before reaching the cortex. More precisely, dorsal root ganglion (DRG) receives information from skin and transmits it to the dorsal column nuclei (DCN). DCN, in turn, transmits information from DRG to the ventral posterior lateral (VPL) nucleus of the thalamus, crossing the midline at the medulla via the medial lemniscus [46]. These relay stations play a key role in stimuli contrast sharpening but we decided to ignore them since we considered that the experimental setup provides enough control over the stimulus and ensures proper sharpness. Hence, the output of all receptors are directly fed to the cortical model (see figure 2).

Skin lesions were made by silencing receptors over a specific area of the skin surface. Instead of transmitting proper values, disabled receptors transmit a null value which more likely corresponds to sensory deprivation. There are three types of lesions (namely type I, II & III) as illustrated in figure 2 These three types correspond to three distinct topological situations. The first type leads to a skin patch that is topologically equivalent to the intact one. The second type introduces a separation of the intact skin patch into two distinct areas and because a stimulus cannot span the two patches at once, these two skin patches are indeed independent. The third type introduces a hole in the topology of the skin and is the most challenging to recover from.

### Cortical Model

A small volume of the primary somatosensory cortex (SI) was modeled using the neural field theory [47]–[50] which considers a given cortical volume 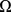 to be a spatial continuum where macro-state variables (such as the mean firing rate) of a population at position 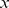 is given by an equation of type:
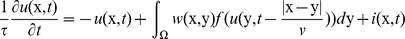
where 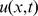 represents the activity (e.g. the membrane potential) at position 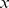 and time 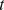, 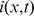 represents the synaptic input, 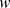 is a weight function measuring the strength of connection between positions 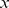 and 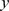, 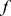 is the firing rate function of a single neuron, 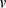 is the velocity of an action potential and 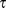 is the temporal decay.

Given the small size of cortical volume, we neglected velocity effects (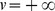, see [5] for a study) and we considered the field to be homogeneous and isotropic, leading to the following simplified equation:
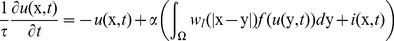
(2)where 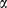 is a scaling factor and 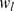 is the weight function of lateral connections. Moreover, we use a simple rectification for the firing function 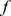 since it is the simplest function that can provide stability for a such field [52], [53]:



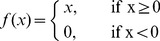
(3)We also considered the input to be a measure of the difference of a given stimulus 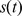 (that correspond to the 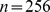 outputs of the skin receptors) with a set of feed-forward weights 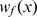 such that for any position **x**, we have:
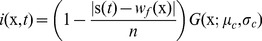
(4)where 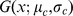 is a corrective Gaussian function. It is corrective in the sense of toric connections since our model does not enforce any toric topology (it is not implemented on a torus). We therefore multiplied 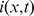 by a fixed-shape Gaussian function in order to correct any kind of boundary conditions side-effect. Throughout simulations the variance of the corrective Gaussian function was 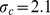, and the mean, 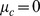. Finally, the lateral connection weight, 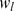, reflects the usual pattern of short-range excitation (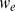) and long-range inhibition (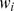):
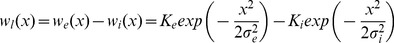
(5)with 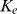, 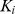 being the amplitudes, 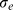, 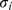 being the variances of the excitatory and inhibitory Gaussian functions respectively, such that generally we consider 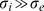.

Cortical lesions were made by silencing units over a specific area of the cortical surface such that dead unit’s activity was always zero. Like for skin lesions, we considered three types of lesions (namely type I, II & III) with same topological properties (see figure 2).

### Neural Population Dynamics

**Figure 3:**
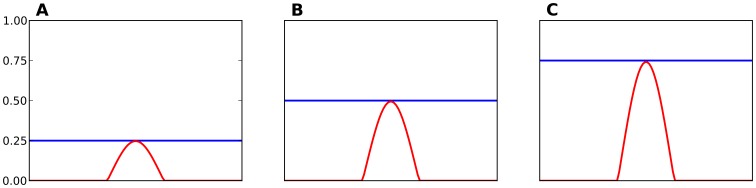
Three solutions of the same one-dimensional neural field. Field response (red curve) for three different uniform inputs (blue curve). In each case, the maximum activity of the field matches the input. The spatial discretization of the field is 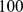 units. **A** Response to input 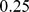. **B** Response to input 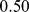. **C** Response to input 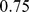.

In his seminal work on neural fields [47], Amari studied the equilibrium solutions, stability and formation of dynamic patterns providing the conditions for such behaviors in the one-dimensional case. Since then, a lot of work has been done in the direction of extending the initial theory to other conditions [48], higher dimensions [49], [54] and different behaviors (see the review by Coombes [50]). In the present work, we are mainly interested in the two-dimensional case since we aim at modeling a cortical sheet. More specifically, we are interested in spatially localized solutions (a.k.a. bumps) that may drive the cortical plasticity and we would like the activity of the field to reflect to some extent a measure of the input, e.g. a measure of the distance between the feed-forward weights of the most activated units (i.e. units from the bump) and the current stimulation. Using a specific set of parameters 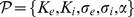 given in table 1, the field can achieve the following property: *for any uniform input 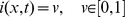, the maximum activity of the field is* 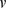. The one-dimensional case is illustrated in the figure 3. Furthermore the same property holds true in the two dimensional case using the same set of parameters, 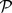.

**Table 1:** Model parameters.

*K_e_*	*K_i_*	*σ_e_*	*σ_i_*	*μ_c_*	*σ_c_*	*σ*	*dt*	*α*	*τ*	*γ*
3.65	2.40	0.1	1.0	0.0	2.1	0.15	0.2	0.1	1.0	0.05

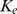
 and 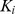 are amplitude of the excitatory and inhibitory weight functions. 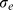 and 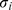 are the variances of excitatory and inhibitory weight functions. The mean and the variance of the corrective Gaussian function are given by 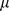 and 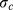, respectively. The variance of stimulus is given by 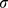 and the mean is variable, although we explain in the text how we compute it. 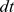 is Euler’s method time step. 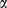 is a constant and 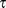 is the time constant of equation (2). 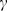 is the learning rate of learning rule given by equation (6).

### Plasticity Rule

A learning rule for the *classical* self-organizing map algorithm [38] has been proposed by Rougier and Boniface [55], where the original time-dependent (learning rate and neighborhood) learning function has been replaced by a time-invariant learning rule. Instead, a dynamic neighborhood function has been introduced that depends explicitly on the distance of the winner to the presented stimulus. On the one hand, if the distance of the winning unit is very close to the presented input, the dynamic neighborhood is rendered very strong but narrow, weakening the learning of other units. On the other hand, when the winning unit is very far from the presented input, the dynamic neighborhood exhibits a very broad but weak pattern, promoting weak learning of every unit in the network. This algorithm has been experimentally proved to be able to achieve self-organization in a similar way as of a regular self-organizing map. Using this idea of a dynamic neighborhood but in the context of neural fields (no notion of a winning unit), we can use the aforementioned *match property* to achieve such behavior.

**Figure 4:**
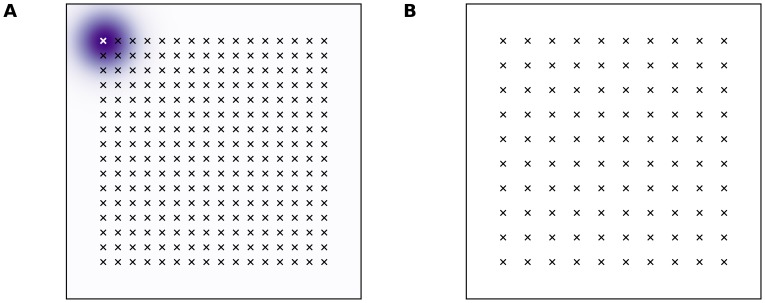
Locations of training and validation stimuli on the skin patch. **A** Training is performed on a set of 16×16 stimuli that are uniformly distributed over the 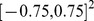 area (skin patch normalized area is 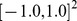) such that any stimulus is entirely located on the skin patch (see example stimulus on upper left corner). **B** Validation (as reported in **C** panels in result figures) is performed on a set of 10×10 stimuli that are uniformly distributed over the 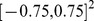 area.

As explained in the introduction, our main hypothesis is that cortical plasticity can be achieved at the level of thalamocortical connections, corresponding to the feed-forward weights in our model. This implies that the network does not need to learn the lateral weights. Therefore, we trained the field using a modified Oja learning rule [56] with the following equation:
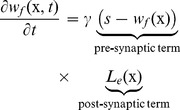
(6)where 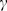 is the learning rate and 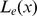 is the total excitation received at the point 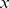 which is given by the two dimensional spatial convolution between the excitatory part of the lateral weight function and the field activity. More precisely, we have:
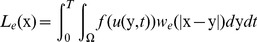
(7)where 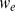 is the excitatory part of the lateral weight function as it is given by equation (5). The idea is to explicitly modulate learning according to the sum of excitation received at a point 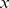 while the inhibition only serves during the competition stage. At this stage, it is important to note that we unified the inhibitory and excitatory neural population into a single population and used positive/negative weights to reflect excitatory/inhibitory action of a neuron onto another. It would be perfectly equivalent to use a dual population but using a single population makes computation faster. In this context, 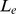 reflects the contribution of excitatory neurons. The learning rule is composed of a pre-synaptic term and a post-synaptic term. The pre-synaptic term reflects the explicit comparison between the stimulus pattern and the pattern of the feed-forward thalamocortical synapse that enter the neuron. This allows to gracefully enforce both Hebbian (LTP) and anti-Hebbian (LTD) learning, controlling the growth of the synapse. The post-synaptic term modulates the pre-synaptic term according to the total sum of excitation (instead of the post-synaptic activity as it would be in the original Oja rule). As illustrated in figure 4, the activation at a specific site and the amount of excitation received at the same site are highly correlated but are nonetheless different: the extent of excitation goes beyond the extent of activation. From a more mathematical point of view, we can also note that 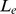 appears to be smooth (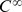) while 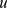 is not (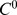). We have started the formal analysis of that property since we think that this makes a critical difference for the self-organization procedure. This signal provides the model with the necessary information on neighborhood topology. Furthermore, the support of 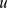 (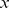 such that 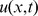 is not null) is constant and independent of the input (this is a property of the neural fields) and it may thus not provide enough information for proper self-organization (we numerically tested a learning rule using 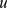 instead of 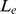 without success). From a biological point of view, this means that a neuron whose membrane potential is below firing threshold may nonetheless learns if it has received enough excitation.

**Figure 5:**
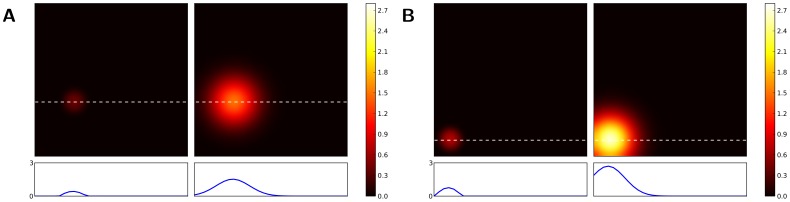
Response of the model and lateral excitation. The response of the model and the amount of lateral excitation at a specific site (center of activity). Plots represent the response profile corresponding to the dashed lines. **A** Before learning. **B** After learning.

At early stage of the training, because of the randomness of the feed-forward weights, any stimulus can cause a weak response of the model at a random place within the field (see figure 5A). As the learning process is ongoing and the feed-forward weights converge according to equation (6), the response of the model becomes stronger and occupies a specific spatial location (see figure 5B).

### Simulation Details

The set of stimuli that is used during training is initially generated by equation (4) over a subset 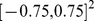 of the skin patch 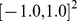. Stimuli locations are set on a regular grid (see figure 4) in order to ensure proper coverage of the patch. During training, a stimulus is uniformly drawn from within this training set. Unless stated otherwise, the same stimuli set is used for all simulations. The neural field has been discretized into 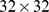 spatial elements and the integration of equation (2) is performed using the forward Euler’s method (time step 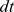 is given in table 1). Feed-forward weights 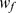 are randomly initialized in the range 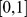. During all simulations we used 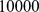 epochs. In each epoch, the stimulus is presented to the model and the field is integrated over a fixed time window while the learning rule is applied to the feed-forward weights. Spatial convolution in equations (2) and (7) is calculated using a fast Fourier transform (FFT) in order to accelerate this operation. Then the activity of the field is reset to zero. This represents the removal of pressure from the skin patch (we could wait for the field to go back to the steady state but it is numerically faster to reset it). The feed-forward weights average evolution 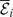 of a neuron 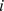 was measured by using the following equation:
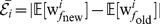
(8)where 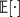 represents the expected value (i.e. the mean value of the array) and 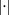 represents the absolute value. Lesions of type I, II and III (skin or cortex) have been implemented using three masks displayed in figure 2. For skin lesion, input was nullified at lesion sites before being transmitted to the neural field while for cortical lesions, lesioned units were nullified at each time step.

The receptive field of each neuron has been computed from a set of 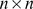 (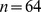 in this work) regularly distributed stimulus 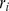 over the subset 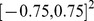 that have been presented sequentially to the model. Each individual neuron activity has been recorded and aggregated into a 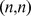 matrix of activities. The size of the receptive field has been identified with the normalized sum of non null values while the center has been computed as the center of mass 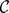 of the receptive field given by the following equation:
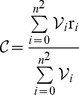
(9)where 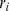 denotes the respective position of stimuli used to compute RF and 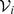 is the activity at position 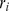. Using self-organization information from the intact model, **we translated those centers** into the skin reference such that topographical information corresponds, this eases the lecture of the figure without changing the results.

Simulations were performed on a HP Z800 Workstation. The source code of all simulations is written in Python (Numpy, Scipy and Matplotlib) and it is available on-line at http://www.loria.fr/~rougier/coding/software/DNF-SOM.tgz. During a simulation of 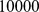 training epochs (sweeps), simulation program consumes ∼190 MB of physical memory and requires ∼13 minutes of CPU time until reaching final epoch.

## Results

For the sake of simplicity we have split figures into 6 panels, starting from the evolution of a RF of the neuron 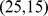 (except the cortical lesion of type II where we used the neuron 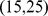) from the epoch number 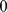 and reaching epoch 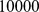 through epochs 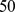, 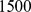 and 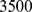. Then we illustrate the preferred location of the neurons by computing the center of mass according to equation (9) and the size of each RF of each neuron in order to depict discs on the skin grid which indicate the preferred location and the size of each RF. In the third panel we illustrate the response of the model to 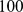 different stimuli, which cover uniformly the 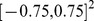 area (see figure 4). In the case of skin lesions the stimuli are larger than the lesion and therefore trigger the neighboring receptors (a single stimulus spans a large portion of the skin). These stimuli are presented to the model and simultaneously the activity of the field is recorded. In the fourth panel, we point out the evolution of the feed-forward weights of the aforementioned neuron and in the two last panels we use two histograms of the size of RFs of the whole neural field. This overall organization allows us to illustrate consistently important alterations that take place during cortical and skin lesions. The histograms were made using 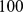 bins. Likewise, we measured the mean and the standard deviation (SD) of the size of RFs before and after cortical lesions and sensory deprivations.

### Emergence of Ordered Topographic Maps

During the early stage of the learning process, the response of the field to a stimulus is not null even though feed-forward weights have been set to random values. It displays instead a localized but weak activity as illustrated in figure 5 A. This is due to neural field properties that guarantee such behavior depending on the amount of lateral excitation and inhibition.

This weak activity bump is highly correlated with the presence of lateral excitation at the location of the former. This allows most active units to learn the presented stimulus proportionally to their lateral excitation (see equation (6)). Once the field has been trained, the response to any stimulus is stronger (see figure 5B) as well as the amount of lateral excitation. This results in an increased learning rate for a stimulus that is already known to the model and thus, it does not change drastically the feed-forward weights anymore. This is a key point of the model since we’ll see in next subsection how this active learning rule may help to recover from lesions.

**Figure 6:**
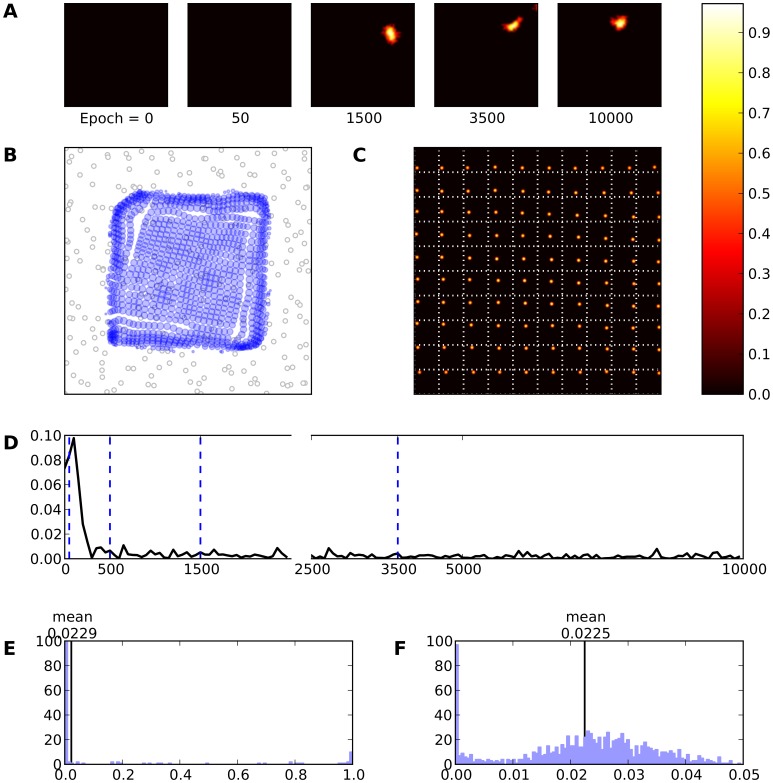
Intact model. **A** Evolution of the receptive field of neuron 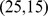 during learning. The neuron is initially silent (epoch 0) but learns quickly to answer to a large range of stimuli (epoch 1500) until finally settling on a narrower range of stimuli. **B** Receptive fields of the whole model. Each blue circle represents a neuron. The center of the circle indicates the (converted) receptive field center and the radius expresses the (relative) size of the receptive field. **C** Response of the model (after learning) to a set of 10×10 regularly spaced stimuli. Each square represent a response to a specific stimulus. **D** This represents the mean evolution of thalamo-cortical weights of neuron 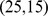 during learning (i.e. 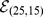). **E** & **F** Histogram of receptive field sizes (100 bins) before (E) and after (F) learning. The final distribution is Gaussian-shaped centered around a mean value of 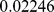. Is is to be noted the high number of very small receptive field size that correspond to neurons on the border of the field that are mostly silent during the whole simulation.

The evolution over time of the RF of the neuron (number 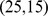) is illustrated in figure 6A. Initially, the neuron is mostly silent, but after 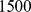, 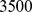 and 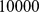 presented stimuli, one can see the development of the RF that is finally precisely tuned to a specific set of stimulus location. This evolution occurs in two phases. In the first phase, the RF is extended and covers a large part of the skin, then in a second phase, as the training process goes on, the RF shrinks and covers only a small part of the skin.

Each neuron now responds preferentially to a specific skin region. Moreover, figure 6C shows the response of the model (after training) to 10×10 different stimuli. It is quite clear that a topographic map has emerged. Each block in this figure represents a response of the model to a specific stimulus (e.g. the block at the upper left corner represents the response of the model to a stimulus at the upper left corner of the skin grid) providing a way to verify self-organization and also provides a frame of reference of the receptive field location on the skin patch. Similar results are illustrated in figure 6B where we used equation (9) in order to compute the location of each receptive field on the skin patch. The radius of each circle has been calculated by using the size of each RF.

**Figure 7:**
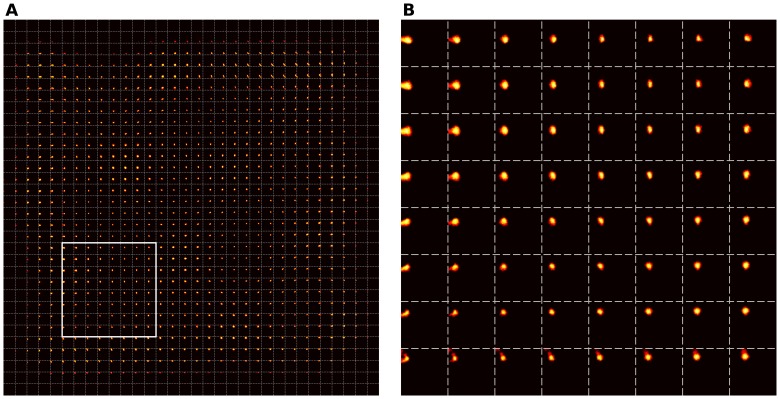
Receptive fields of the intact model. **A** Of the whole cortical sheet. **B** Magnification of the white box.

In addition, the distribution of RF sizes can undergo alterations during training. Panel 6E shows the distribution of RF sizes before any learning occurs in the model. As one can expected, there is no RFs at all since the neurons have not yet learned anything. However, once learning is finished, one can see in figure 6F the normal-like distribution of the RF sizes. There is a high-value component near zero which indicates a large number of very small RF sizes that is due to side (border) effects of the neural field. Other RF sizes follow a normal-like distribution with mean 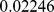 (SD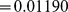). This indicates that there is a better acquisition of RFs at the center of the field than at the periphery. Combining all the aforementioned results we can conclude that the model has achieved proper self-organization. Subsequently, the emergence of such an ordered map tends to confirm the initial hypothesis that thalamocortical connections are an adequate site of plasticity for both the formation and the maintenance of topographic representations. In this context, lateral connections mainly serve as support for competition at the cortical level for the emergence of a unique bump of activity that drives learning. Finally, figure 7 displays the RFs of all the neurons after 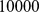 presented stimuli. One can clearly see that ordered representations have emerged over the whole field.

### Reorganization after a Skin Lesion

**Figure 8:**
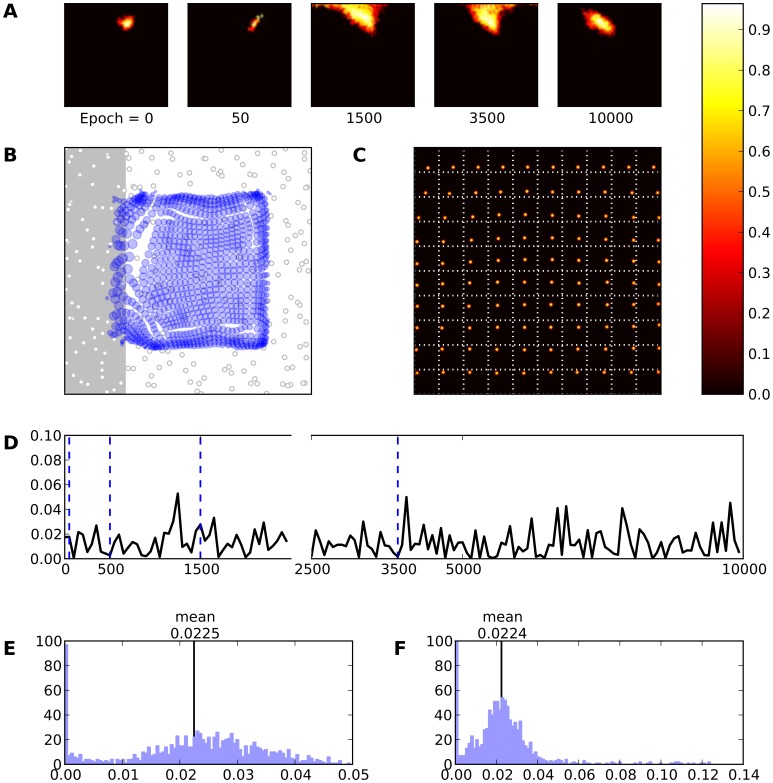
Skin lesion type I (gray area). **A** Evolution of the receptive field of neuron 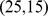 during retraining after a skin lesion of type I. **B** Receptive fields of the whole model. **C** Response of the model (after retraining) to a set of 10×10 regularly spaced stimuli. **D** This represents the mean evolution of thalamo-cortical weights of neuron 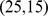 during retraining (i.e. 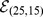. **E** & **F** Histogram of receptive field sizes (100 bins) before (E) and after (F) skin lesion. The initial distribution is Gaussian-shaped centered around a mean value of 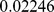. However, the final distribution is a Poison-like centered around a mean value of 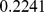 with a long tail indicating that there are a lot of neurons whose RFs have underwent an expansion. At the same time an almost equivalent amount of neurons has moved toward smaller RF sizes underlying that a shrinkage of RFs has taken place.

**Figure 9:**
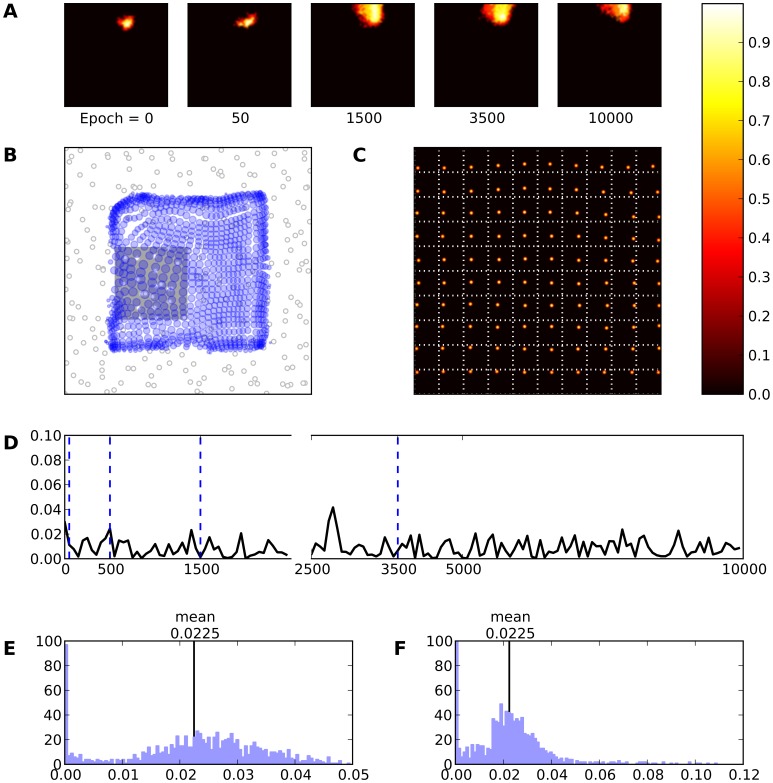
Skin lesion type III (gray area). **A** Evolution of the receptive field of neuron 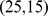 during retraining after a skin lesion of type III. **B** Receptive fields of the whole model. **C** Response of the model (after retraining) to a set of 10×10 regularly spaced stimuli. **D** This represents the mean evolution of thalamo-cortical weights of neuron 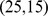 during retraining (i.e. 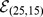. **E** & **F** Histogram of receptive field sizes (100 bins) before (E) and after (F) skin lesion. The initial distribution is Gaussian-shaped centered around a mean value of 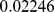. Although, the final distribution is a Poison-like centered around a mean value of 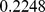 with a long tail indicating that there are a lot of neurons whose RFs have underwent an expansion. At the same time an almost equivalent amount of neurons has moved toward smaller RF sizes underlying that a shrinkage of RFs has taken place.

**Figure 10:**
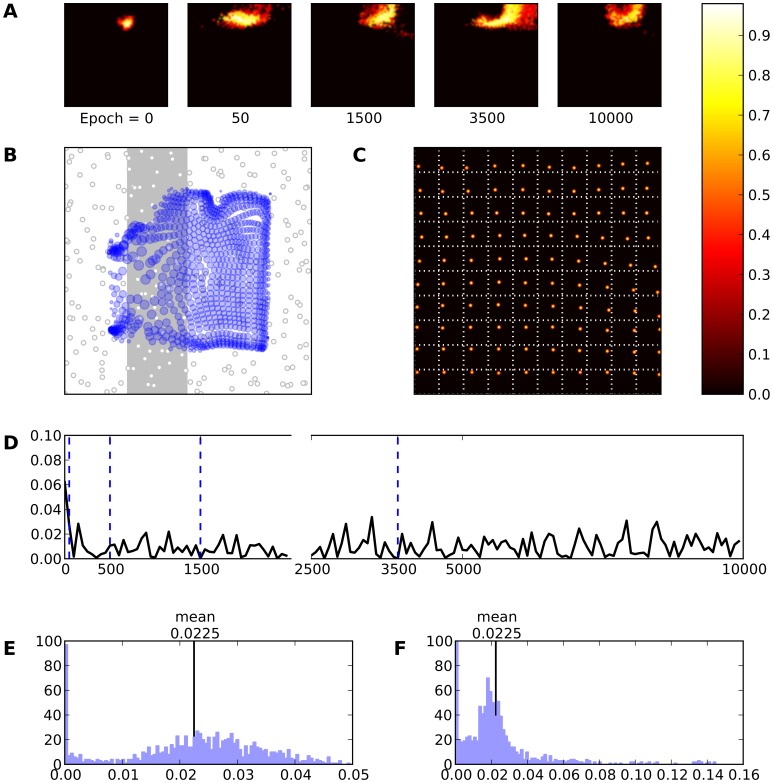
Skin lesion type II (gray area). **A** Evolution of the receptive field of neuron 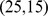 during retraining after a skin lesion of type II. Immediately following skin lesion (epoch 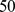), RF tends to expand. This phenomenon persists until the final epoch is reached where a shrinkage takes place. **B** Receptive fields of the whole model. **C** Response of the model (after retraining) to a set of 10×10 regularly spaced stimuli. **D** This represents the mean evolution of thalamo-cortical weights of neuron 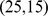 during retraining (i.e. 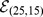). **E** & **F** Histogram of receptive field sizes (100 bins) before (E) and after (F) skin lesion. The initial distribution is Gaussian-shaped centered around a mean value of 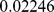. However, the final distribution is a Poisson-like centered around a mean value of 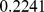 with a long tail indicating that there are a lot of neurons whose RFs have underwent an expansion. At the same time an almost equivalent amount of neurons has moved toward smaller RF sizes underlying that a shrinkage of RFs has also taken place.

We first studied the case of sensory deprivation resulting, for example, from damaged sensory nerves or physical damages to the receptors. This has been modeled by silencing a specific amount of skin receptors (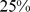 in type I and type II skin lesions and 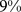 for type III skin lesion) such that only a subpart of previously sensory information is made available to the cortex. A lesion was made onto three specific areas which are referred as lesion type I, type II, and type III (see figure 2 for precise shape). We only report, in this section, results from type II lesion since we found qualitatively equivalent results regarding type I and type III lesions (see figures 8 and 9, respectively, for details). Following a skin lesion, the model has been retrained over 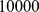 epochs using the same set of stimuli as before but with missing values from disabled receptors. Panel 10A shows the temporal evolution of the receptive field of unit 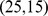 and figure 10C shows the overall reorganization of representations that has occurred according to the response of the model to 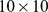 different stimuli. This is most clearly illustrated in figure 10B that displays the preferred location of units that do not intersect with the skin lesioned area. Comparing the RFs illustrated in this figure with the ones on figure 6B, one can conclude that the sizes of RFs which were previously innervated by the lesioned skin area are now larger. This is because neurons lost their preferred input and therefore the balance of excitation and inhibition is disrupted. Therefore neurons expand the size of their RFs in order to acquire new inputs. This resilient behavior can be easily explained because thalamus provides divergent inputs to the cortex. Neurons that were previously tuned to dead receptors will expand their RFs in order to reach neighboring receptors. This expansion takes place immediately after the sensory deprivation as shown in figure 10B and 10A where the RF of neuron 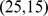 underwent an expansion immediately after sensory deprivation (epoch 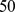). Panels 10E and 10F shows the histograms before and after sensory deprivation, respectively. The former corresponds to the intact model which we discussed in previous subsection and the later corresponds to the sensory deprivation case after retraining of the model. There is a small shift of the main peak of the distribution from the value of 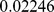 towards 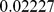, but with a noticeable spreading of the RFs (SD = 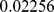) size indicating a new distribution of RF towards both smaller and larger receptive fields (while the large component at zero because of border effects remains). This alteration in the distribution tends to show that even if most RFs have shrunk, a significant portion have expanded in size.

Furthermore, from figure 10A describing the temporal evolution of unit 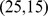, we can see that reorganization occurs in two major phases. At the beginning, each neuron innervated by deprived skin area undergoes an expansion of its RF simultaneously with a spatial shifting in order to capture a new skin area (first phase). This lasts almost during the whole retraining process. Near the end of training process the affected neuron has shrunk its RF (second phase). Similar to this finding, Foeller in [21] proposes a three-phases model of the RFs reorganization. In the first phase and due to reduction of inhibitory connections the RFs expand their size. During the second phase, a further increase of RFs size is taking place because of homeostatic plasticity of GABA circuits. Finally, in the third phase, a shrinkage of RFs around their new centers occurring as it is driven by re-established inhibitory connections. We can merge the two first phases into one in our model since we do not involve any kind of neurobiological mechanism and therefore a such detailed timescale is not necessary.

However, the refinement of the RFs is not so exquisite because, according to our main hypothesis, lateral connections remain fixed and non-plastic throughout all simulations. This means that neurons are able to receive proper excitation and inhibition conserving the competitive nature of the reorganization process. Nevertheless, a better refinement could be possible by using a learning rule also for the lateral connections. Furthermore and as it has been explained, the precise type of lesion does not impact result in a significant way. Neurons that were preferentially tuned to a disabled skin area tends to have their receptive fields shifting away from the site of the lesion to neighboring locations. However, for type II and type III lesions, there is an additional topological constraint onto those neurons because they can still be part of an active bump in the field (and tune their receptive field accordingly). They can be thus attracted either to the left or to the right part of the lesion site for type II and to any border of the lesion site for type III. This explains that some neurons do not express any kind of resilience and have their preferred location still on the lesioned area even after extensive retraining (figures 10B and 9B). This also explains the increased oscillations in average evolution of feed-forward weights, 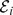 (figures 10D and 9D).

### Reorganization after a Cortical Lesion

**Figure 11:**
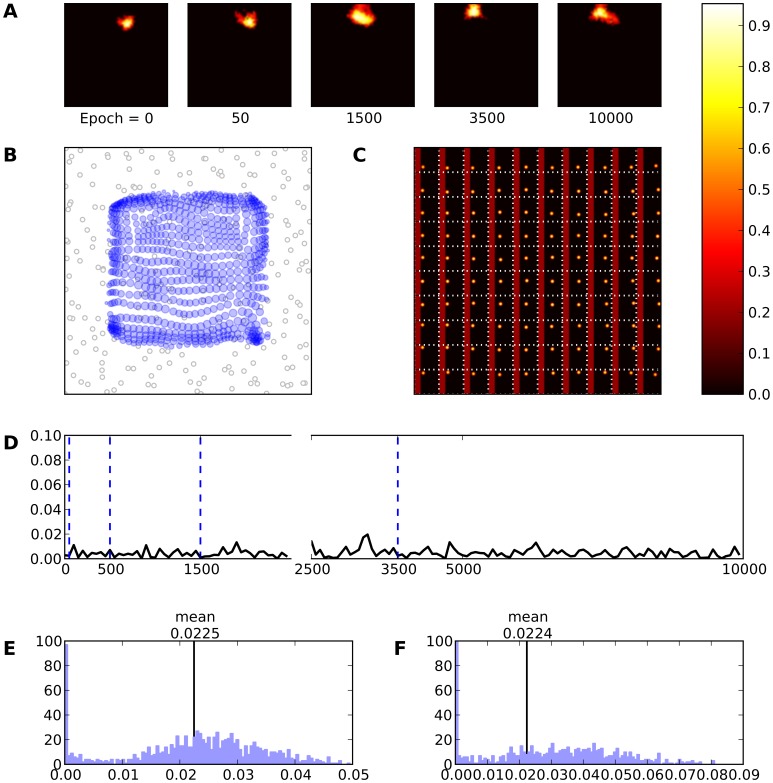
Cortical lesion type I (red area). **A** Evolution of the receptive field of neuron 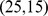 during retraining after a cortical lesion of type I. Immediately following the lesion (epoch 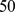), RF tends to expand. This phenomenon persists until the final epoch is reached. **B** Receptive fields of the whole model. **C** Response of the model (after retraining) to a set of 10×10 regularly spaced stimuli. The activity of the model is now bound to the unlesioned area. **D** This represents the mean evolution of thalamo-cortical weights of neuron 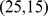 during retraining (i.e. 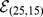). **E** & **F** Histogram of receptive field sizes (100 bins) before (E) and after (F) skin lesion. The initial distribution is Gaussian-shaped centered around a mean value of 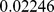. However, the final distribution is a uniform-like centered around a mean value of 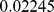 (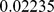). This uniform-like distribution indicates the existence of neurons whose RFs have underwent an expansion, but not a shrinkage.

We also addressed the case of reorganization by causing a cortical lesion, i.e. silencing some neurons in the neural field. In living tissue, such damages can be caused by a stroke, a hematoma or by a surgery either for therapeutic or experimental purposes. Subsequently, we caused three different types of cortical lesions (i.e. type I, II and III) by applying a mask to the self-organized representational map as we previously described in methods section. These lesions were of an extent of 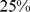 of the total amount of neurons. We applied a type I lesion close to the border of cortical sheet, a type III, localized ablation and a type II band-shaped lesion in the mainland of cortical sheet (see figure 2 for precise lesion shapes). Thus, after retraining of the network using 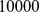 stimuli patterns for each of these lesion cases, a new representational map has emerged as it is depicted in figure 11 for type I lesion.

Comparing the RFs size before and after cortical lesion, they have been clearly altered. After lesion, RFs tend to become larger and consequently to respond to larger skin areas. More precisely, RFs size after lesion is almost twice bigger compared to pre-lesion ones. As it is shown in figure 11A the evolution of neuron 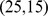 after a cortical lesion of type I has been altered. Immediately following the lesion (epoch 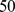), RF has expanded itself and cover the skin patch which was previously represented by lesioned neurons. The temporal evolution of the RF indicates that this neuron has changed its preferred input in order to promote the recovery. In addition and as it is illustrated in figure 11B, RFs of almost all neurons have been changed. The radii of the blue discs have been increased in size, especially around the lesion site. Taking also into account the results coming from the histograms of figures 11E and 11F concerning the pre-lesion and the post-lesion cases, respectively, one can see the overall distribution of RFs size has changed in favor of a larger number of large RFs. The mean value of RFs after cortical lesion is equal to 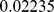 and the SD is equal to 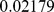 indicating a significant spread of RF sizes.

**Figure 12:**
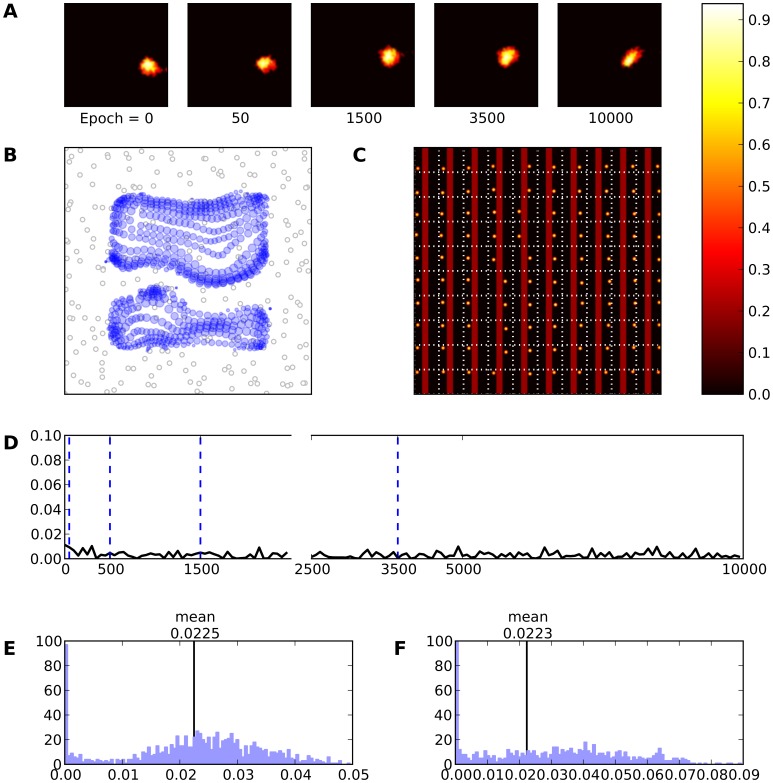
Cortical Lesion type II (red area). **A** Evolution of the receptive field of neuron 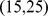 during retraining after a cortical lesion of type II. This particular neuron has not expanded its RF but it has replaced its preferred location as it is depicted at the final profile (epoch 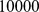). **B** Receptive fields of the whole model. The cortical lesion is appeared at the preferred locations since the previously corresponding neurons are now affected by the lesion. The RFs around the lesion have been increased in size comparing with the corresponding pre-lesion figure 6**B**. **C** Response of the model (after retraining) to a set of 10×10 regularly spaced stimuli.The activity of the model is now bound to the unlesioned area. **D** This represents the mean evolution of thalamo-cortical weights of neuron 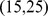 during retraining (i.e. 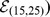. **E** & **F** Histogram of receptive field sizes (100 bins) before (E) and after (F) skin lesion. The initial distribution is Gaussian-shaped centered around a mean value of 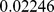. However, the final distribution is a Uniform-like centered around a mean value of 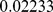. This uniform-like distribution indicates the existence of neurons whose RFs have underwent an expansion, but not a shrinkage as in cortical lesion type I case. In this case we illustrate results regarding neuron 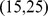 because neuron 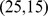 lies in the lesion.

**Figure 13:**
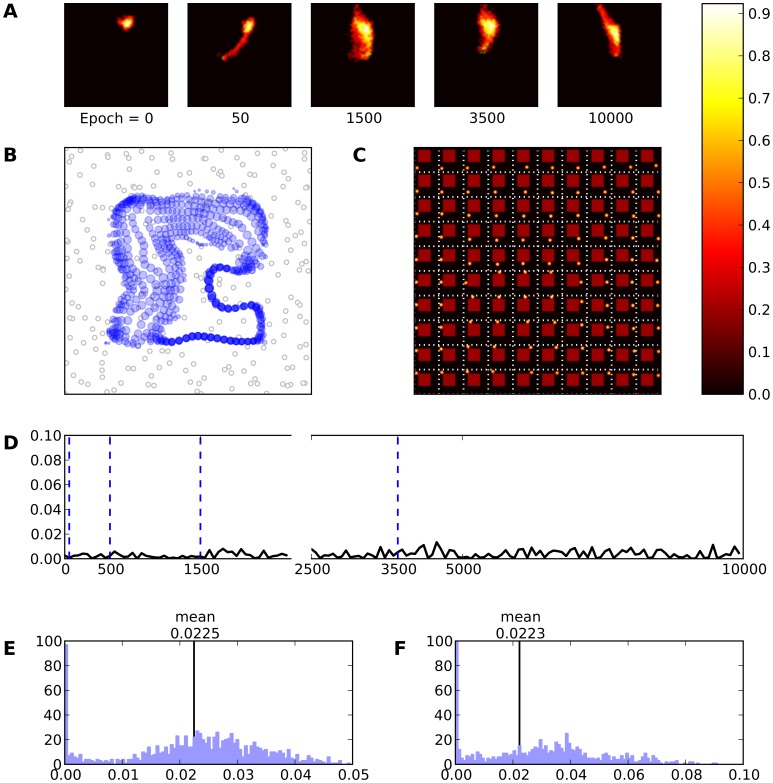
Cortical lesion type III (red area). **A** Evolution of the receptive field of neuron 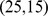 during retraining after a cortical lesion of type III. This particular neuron has expanded its RF immediately after lesion and moreover it has has replaced his preferred location as it is depicted at the final profile (epoch 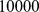). **B** Receptive fields of the whole model. The cortical lesion is appeared at the preferred locations since the previously corresponding neurons are now affected by the lesion. The RFs around the lesion have been increased in size comparing with the corresponding pre-lesion figure 6**B**. **C** Response of the model (after retraining) to a set of 10×10 regularly spaced stimuli. **D** This represents the mean evolution of thalamo-cortical weights of neuron 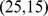 during retraining (i.e. 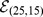). **E** & **F** Histogram of receptive field sizes (100 bins) before (E) and after (F) skin lesion. The initial distribution is Gaussian-shaped centered around a mean value of 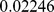. However, the final distribution is a Uniform-like centered around a mean value of 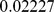. This uniform-like distribution indicates the existence of neurons whose RFs have underwent an expansion, but not a shrinkage as in cortical lesion type I case.

This is quite consistent with Sober [12] who reported similar results with the noticeable difference, that weeks after a lesion, cortex is able to completely recover, having its neurons RFs sharpened. This is because of re-establishment of inhibitory connections and/or sprouting of neural axons as it has been proposed by Florence [57]. Consequently, the refinement of RFs arise in two phases. During the first phase, there is an expansion of RFs towards lost territories followed by a shrinkage of the second phase. In our computational experiments there is no such shrinkage during the second phase because of the fixed set of lateral connections as it is depicted in figures 11A, 12A and 13A. This leads us to ascertain that the lateral connections are crucial to the development of stable representational maps. Neurons are not able to precisely refine their RFs since there is no balance mechanism between excitation and inhibition within cortical circuits. Sur [58] has shown that intralaminar excitatory connections are the major factor for expansion of RFs. In consequence, RFs in figures 11A and 11B have successfully expanded themselves leading to larger skin area representation but have failed at shrinking themselves because of the non-plastic lateral connections. Furthermore, it is remarkable to see that neurons have migrated to cover the whole skin surface again (figures 11A, 11B) and non-functional representations (just after lesion) have been *recaptured* by neighboring units. The model is able to respond again to stimuli applied on areas innervating neurons within the lesioned cortical area (figure C). This indicates that other neurons took over and recovered from lesion by migrating their representations towards the lost ones, making the cortical patch functional but degraded.

Recovery from cortical lesions of type II and III is displayed in figures 12 and 13 and show the degraded response of the model with only a partial recovery of lost territories. Furthermore, figures 12A, 12B and 13A, 13B clearly show that most RFs have been shifted and expanded spatially without any kind of refinement except for a small number which have underwent a shrinkage as it is pointed out by figures 12 F and 13F. This behavior can be explained quite simply in terms of topology. The first type of cortical lesion is topologically equivalent to the intact one while type II lesion introduces a separation of the cortical patch into two distinct patches and type III introduces a hole in the topology. In both cases, neurons from either sides of the lesion cannot cooperate because their influence is mostly inhibitory (due to their respective distance from each other). This means that the active population resulting from the competition cannot exist on any borders of the lesion. This brings severe constraints to the self-organization process that can only be partially overcome without relearning a new topology through the modifications of lateral connections.

## Discussion

We have introduced a computational model of primary somatosensory cortex that is able to develop topographic maps, maintain and reorganize them in the face of lesions. We used neural fields as a mathematical and computational framework and focused on area 3b innervated by hand mechanoreceptors. The combination of such neural field with a simple Hebbian/anti-Hebbian like learning rule advocates for an unsupervised, distributed, robust and biologically plausible model of a (simplified) somatosensory cortical model where thalamocortical connections are the main sites of plasticity. The major finding of our model is that a topographic map can emerge as a consequence of the interaction between thalamus and cortical excitatory afferent connections. These feed-forward connections are capable of causing the reorganization of a topographic map even in the presence of a cortical lesion or a sensory deprivation. Bruno in [34] has shown that excitatory thalamocortical connections can synchronize themselves in order to drive cortical neurons without making use of any kind of cortical amplification mechanism. This enhanced our hypothesis, which states that the main effort of the emergence and reorganization of a topographic map can be promoted by thalamocortical connections. This also holds for all three investigated cases. First, the formation and emergence (one) of a topographic map. Second, sensory deprivation (two - congenital and contracted). And in the end, cortical lesions (four - congenital, boundary contracted, centered contracted, localized ablation).

Those results are quite consistent with the existing literature on the computational modeling of the somatosensory cortex even though we think we brought new insights on the inner mechanisms. One the earliest model of the SI has been proposed by Pearson et al. [41]. They designed a computational model of the somatosensory cortex based on a dual population of neurons (excitatory/inhibitory) which receive topographic projections from two receptor sheets corresponding to the glabrous and dorsal surfaces of the hand. Following the repetitive tapping of the sensory surface, the model is able to shape itself into several segregated neuronal groups that are dedicated to a subpart of the whole sensory space. Authors used both intrinsic and extrinsic connections modifications although they underlined that they do not know if this is really the case *in vivo*. Joublin et al. [59] introduced a combination of neurophysiological recordings from rats with computational simulations. The model is described using a set of Wilson-Cowan equations and the architecture is made of three layers (receptors grid, subcortical grid and cortex). Authors tried to keep the model close to real data in order to be able to compare their simulations with neurophysiological data. However, the model relies on a pre-cortical level whose role is to topographically order representations in order to simplify the simulation. Furthermore, they didn’t treat lesion cases and focused instead on the learning rule pointing out that different learning rules underlying different forms of plasticity. Xing and Gerstein [60]–[62] used a spiking neural network within a three layer model (i.e. receptors, thalamus, cortex) paying attention to the lateral connections. Authors showed that inhibitory connections are crucial for limiting the number of activated cortical neurons, while the balance between excitation and inhibition is crucial to the stability of the network. This is quite consistent with our own results since the competitive process occurring within the model relies on a precise balance between inhibition and excitation. In the cortical lesion cases, we’ve also shown that the model cannot achieve full recovery without modifications of the lateral connections. This is again quite consistent with the important role of lateral connections given by previous models even though those models did not address specifically the case of cortical and cutaneous lesions. We can conclude with them that lateral connections play an important role and the refinement of RFs following a lesion may be due to the modification of lateral connections. However, we maintain that thalamocortical afferent connections are the main sites of plasticity for both primary self-organization and later reorganization.

We would like now to point out a few more interesting results. Foeller and Feldman [21] as well as Florence et al. [63], proposed that RFs are capable of refinement and shrinkage during a long-term reorganization process of a topographic map in the presence of a sensory deprivation. Our model, due to non-labile lateral connections, is not able to achieve such precise refinement during the reorganization of the topographic map. This leads us to claim that lateral connections is a major moderator of RFs, especially during the reorganization process of the cortical sheet. Nevertheless, we have been able to show the expansion of RFs, which means that RFs are able, during the reorganization process, to represent a larger skin area rather than they did before lesion. But this is only one part of the whole picture as it is only one out of two (or maybe three) reorganization phases. This second phase is missing in our model. During that reorganization phase a shrinkage of RFs takes place due to adaptation of lateral connections, the sprouting of new intra-cortical connections and the left-over unaffected thalamocortical connections. In addition lateral connections must be an important and valuable mechanism of the balance between excitatory and inhibitory neural populations, which, in turn, steers to the reorganization of robust topographic maps. Nevertheless, our model indicates that even without relearning lateral connections, cortical sheet is able to fully or partially recover from a cortical lesion depending on its type. Lesion of type I does not modify the topology of the field and allow for a robust and full recovery while lesions of type II and III are more problematic and leads to partial recovery only. From a neurophysiological point of view such cortical lesions means that the skin patch which provides afferent input to the dead neurons loses its cortical representation. Hence, neurons that are unaffected by lesion receive input from the non-representative skin patch and a reorganization of the SI topographic map takes place. The consequence in our model is an expansion of the RF for the unaffected neurons (see figure 13B). However, one would expect unaffected neurons to cover almost the whole skin patch or at least a larger part. Instead, it is obvious that there is a covering of the skin patch but still there is a part of the skin which remains uncovered. This means that there is input from some areas of the skin but the responsible neurons are now dead. After the reorganization process a new topographic map has formed and hence the unaffected neurons have taken over the previously non-representative territories of the skin patch. This phenomenon is illustrated in figure 13C, where the model is able to respond to different stimuli. Therefore, the tuning of RFs of unaffected neurons is not optimal. We believe that this is closely related to the lack of relearning of lateral connections. More precisely, the cortical lesion disrupts the balance in the lateral connections and we do not allow the model to *fix it* by relearning these connections. This seems to be a critical process because lateral connections are not able to convey proper competition anymore.

**Figure 14:**
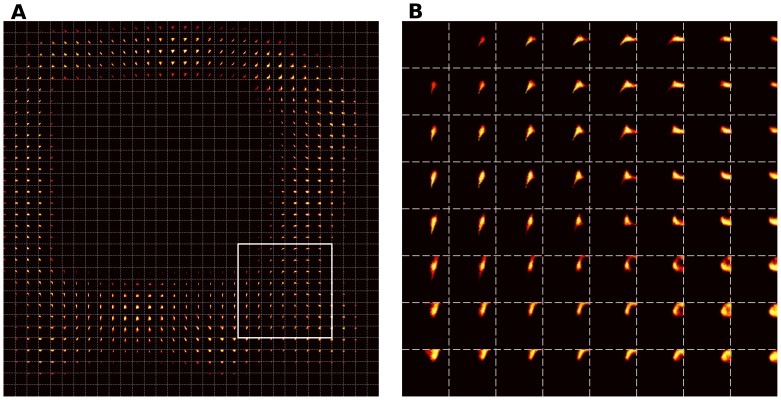
Receptive fields of model after a type III cortical lesion. A RFs of the whole cortical sheet and B a magnification of surrounded, by the white box, area.

At this point, we can point out two major characteristics of a cortical lesion (or ablation) that could be responsible for a proper reorganization and recovery of a cortical sheet. First, is the location of the lesion, *Where the lesion is located?* and second the extent of the lesion, *What is the amount of the dead neurons?* Both, location and extent are intertwined, in a fashion that the former pervades the later and *vice versa*. Therefore, we can discriminate two different cases. First, if the lesion is located around the border of two or more cortical representations and provided the extent of the lesion is not too large, then recovery is easily achievable. This is because of the large amount of left over neurons and afferent connections. However, if the extent of the lesion is large enough, then the representations cannot recover completely and they may even not recover at all. Second, if the lesion is located within a single representation, recovery is only a matter of the extent of the lesion itself. That is because, if a significant amount of neurons are affected by the lesion, there is no enough neurons to deploy their RFs and revive previously lost territory. Yet, in a localized lesion case we noticed that in the vicinity of the lesioned area, there are some neurons which do not respond so intensively as the others and those neurons drive other neurons leading them to reorganize themselves. As it is depicted in figure 4 those neurons have a specific shape which is inherited by the neighboring neurons that expand their RFs omnidirectionaly. Similar findings have been pointed out by Sober in [12], where a disinhibitory halo around cortical ablation has been found. Proximal neurons to this halo are able to drive the reorganization of the neighborhood neurons via their intact lateral connections. Proximal neurons have been loosed their inhibitory connections due to ablation and therefore they have omnidirectionally expanded RFs. Furthermore distant neurons have narrower RFs. Likewise, the case of cortical lesion type III of our model presents a similar behavior as it has been illustrated by figures 6B and 13B considering the pre- and postlesion state, respectively. RFs in the later figure are larger than those in the former figure. In the later figure neurons around lesion have larger RFs in size. This is in accordance with the results of Sober and the so-called disinhibitory halo. Although, in our model we keep the lateral connections fixed and therefore in the case of a cortical lesion there is no way to recover them. Hence, a disturbance of lateral connections triggers a disturbance of excitation/inhibition balance and thus neurons close to lesion receive mostly excitatory connections rather than inhibitory, which in turn causes the expansion of the RFs around lesion and the shrinkage of the distant ones. To test further this hypothesis, we can observe that lateral cortico-cortical connections are a major component of the competitive mechanism that allows to have a unique and compact active population. The shape of this population is critical for learning since it enforces the topology within the model. More precisely, we can predict that any modification on the size of the active population would have a direct impact on the receptive fields. For example, if we were to decrease the inhibition level at the cortical level while blocking learning, the size of the active population would grow and this would result in larger receptive fields. This would mean a loss in sensory representation: the two point discrimination distance would be increased. On the opposite, if we were to increase the inhibition level, the size of the active population would become smaller and lead to smaller receptive fields (and higher precision).

The model has been kept deliberately simple and it comes as no surprise that a number of known mechanisms have not been taken into account like for example homeostatic mechanisms and/or metaplasticity which have been proposed by Turrigiano and Nelson [64] as moderator factors of lateral connections. The former conserves and regulates the average activity of brain circuits by scaling neural synapses and the later prevents them from saturation effects [65]. As future work we left the examination of homeostatic mechanisms and metaplasticity as we believe that this model is offered for further investigation through its ability to adjust its activity depending on the intensity of stimulus. This, in turn, can prevent networks from saturation effects in the same way metaplasticity may affect neural circuits of the brain. Another aspect we did not treat is the phenomenon of spontaneous activity. It is rational to discuss about this because it seems to play a key role in the development of a topographic map within cortex. For instance, Katz and Shatz [66] and Khazipov and Luhmann [35] have found a mechanism which could explain the early formation of L4 in barrel cortex and V1 in rats, respectively. In both cases the topographic map has been formed almost completely before birth. Adding to that findings from Khazipov et al. [36], we can conclude that a fetus *in utero* may take advantage of spontaneous movements in order to establish an early formed topographic map in L4 within a sensorimotor loop. We also neglected top-down mechanisms such as attentional modulatory signals. Knight et al. in [67] have proposed that the prefrontal cortex acts as a modulator of balance of excitation and inhibition of the brain. This provides a straight-forward attentional mechanism since this regulation of balance can affect the receptive fields of neurons. Furthermore, according to the results of Schaefer [68], prefrontal cortex seems to provide to somatosensory cortical areas a gating mechanism which is able to refine receptive fields through inhibition/excitation regulation regarding to attention. We neglected such mechanisms in this work because we believe that they are out of the scope due to the lack of a closed loop (e.g. sensorimotor loop). We thus left as future work the investigation of the role of top-down mechanisms in topographic maps formation and reorganization.

In conclusion, even though this model does not consider all neurophysiological aspects which might play an important role in the overall organization process, we believe that it can help to investigate further the emergence of somatotopic maps during the early months of life. The model is simple enough from a mathematical/computational point of view to allow for further refinement that could potentially give account on more experimental data.
